# Recent Advances in the *Trichomonas vaginalis* Field

**DOI:** 10.12688/f1000research.7594.1

**Published:** 2016-02-11

**Authors:** David Leitsch

**Affiliations:** 1 Institute of Parasitology, Vetsuisse Faculty of the University of Bern, University of Bern, Längassstrasse, Bern, 3012, Switzerland

**Keywords:** Trichomonas vaginalis, trichomoniasis, non-viral sexually transmitted disease, hydrogenosome, metronidazole

## Abstract

The microaerophilic protist parasite *Trichomonas vaginalis* is occurring globally and causes infections in the urogenital tract in humans, a condition termed trichomoniasis. In fact, trichomoniasis is the most prevalent non-viral sexually transmitted disease with more than 250 million people infected every year. Although trichomoniasis is not life threatening in itself, it can be debilitating and increases the risk of adverse pregnancy outcomes, HIV infection, and, possibly, neoplasias in the prostate and the cervix. Apart from its role as a pathogen, *T*. *vaginalis* is also a fascinating organism with a surprisingly large genome for a parasite, *i*. *e*. larger than 160 Mb, and a physiology adapted to its microaerophilic lifestyle. In particular, the hydrogenosome, a mitochondria-derived organelle that produces hydrogen, has attracted much interest in the last few decades and rendered *T*. *vaginalis* a model organism for eukaryotic evolution.

This review will give a succinct overview of the major advances in the *T*. *vaginalis* field in the last few years.

## Introduction

*Trichomonas vaginalis* (Tv) is a globally occurring anaerobic/microaerophilic protist parasite which colonizes the epithelium of the human urogenital tract. Although often asymptomatic, Tv infections can cause inflammation in the cervix, the vagina, and the urethra. Based on estimates of the World Health Organization (WHO) from 2008 ^1^, trichomoniasis constitutes the most prevalent non-viral sexually transmitted disease (STD) worldwide, affecting more than 276 million people every year. Women and men are infected with comparable frequency, but in men symptoms are normally mild and infections are cleared by the host’s immune system within weeks. In women, however, Tv infections can persist for many years, and symptoms, mainly pruritus caused by inflammation and odorous vaginal discharge, can attain a severity which is debilitating. As trichomoniasis is not a life-threatening disease, it was often belittled as a “nuisance infection” in the past. A large number of studies from the last 20 years or so, however, have shown that underlying Tv infections increase the risk of adverse pregnancy outcomes and contagion with HIV virus ^2^. Given the fact that HIV and Tv are often jointly epidemic in many parts of the world, this is an alarming finding. Moreover, a higher risk of developing prostate cancer due to Tv infection has been suggested ^2^.

Before the development of the 5-nitroimidazole drug metronidazole in 1960, trichomoniasis was notoriously difficult to treat. Nowadays, most patients can be successfully treated with metronidazole or another more effective 5-nitroimidazole derivative, tinidazole. However, resistance to 5-nitroimidazoles does occur and seems to be on the rise ^3^. In addition, allergic reactions to nitroimidazoles have been reported and side effects of nitroimidazole treatment can be disturbing.

Apart from its role as a pathogen, Tv has attracted great interest from geneticists, biochemists, and evolutionary biologists after the discovery of the hydrogenosome ^4^, a mitochondrion-like organelle which generates hydrogen. Due to its microaerophilic lifestyle, Tv does not have the ability to generate ATP by oxidative phosphorylation but depends on substrate-level phosphorylation. Originally, it was assumed that the hydrogenosome is an ancestral form of the mitochondrion ^5^, which kindled interest in Tv as an assumed archaic eukaryote. This notion, however, has been thoroughly revised after publication of the Tv genome in 2007 ^6^. It is now apparent, based on phylogenetic studies, that the hydrogenosome constitutes a reduced form of fully developed mitochondria. Nevertheless, from the evolutionary and cell biologist’s point of view, the hydrogenosome remains an intriguing organelle, and the extraordinary size of the Tv genome, exceeding 160 Mb, will certainly provoke further research in the years to come.

In this review, I will give a brief but comprehensive overview of the advances in the research on Tv from the last 5 years or so, spanning from the epidemiology to the infection biology, treatment, and cell biology of this fascinating parasite.

## Epidemiology

Although Tv is a worldwide occurring parasite, prevalence rates differ very strongly in different parts of the world. In the Americas, for example, its incidence is calculated to be as high as 180 per 1000 men and women, whereas in South-East Asia estimates are much lower, with 40 to 50 per 1000 men and women ^7^. In total, 276 million infections with Tv are believed to occur worldwide and *per annum* ^1^. These numbers are very high indeed, but it is estimated that most Tv infections (up to 80%) are asymptomatic ^8^. Importantly, men are infected equally frequently, but 89% of trichomoniasis cases are actually diagnosed in women because of their higher incidence of symptoms, which are sometimes severe and debilitating. The main concern about Tv infections, however, is their predisposing nature for other diseases or sequelae ^2^, and a number of new studies give justification to this concern. For example, Tv was found to be associated with human papilloma virus infections and cervical cytological abnormalities ^9^. Moreover, in a meta-analytical study, strong statistical evidence was presented for an association of an underlying Tv infection and preterm birth ^10^. Most importantly, however, evidence for a predisposition for infection with HIV in Tv-infected individuals is mounting. In a meta-study on 31 studies, it was concluded that the risk of HIV acquisition is increased 2- to 3-fold in Tv carriers ^11^, and it was found that Tv infection increased the risk of HIV infection 2.5-fold in macaques, which serve as a non-human primate model. Accordingly, it was calculated that annual screening for Tv would save US$553 per woman and lifetime in the prevention of new HIV infections to susceptible male partners in the United States alone ^12^.

In order to get a picture of the genetic diversity of Tv, a large-scale study ^13^ was conducted, subjecting 235 Tv isolates, collected from all around the globe, to microsatellite genotyping ^14^. Intriguingly, Tv was found to group into two distinct clusters or “types”. Both types occur worldwide with comparable frequency, although type 1 is presumably the older clade ^13^. Interestingly, the presence of Tv ** virus (TVV) is unequally distributed within the two types, with about 70% of all type 1 isolates, but only 30% of type 2 isolates, carrying the virus. Conversely, metronidazole resistance is far more prevalent in type 2 isolates.

## Treatment

Since 1960, metronidazole and other 5-nitroimidazoles, such as tinidazole, have been the mainstay of Tv treatment ^3^. 5-nitroimidazoles have been reported to damage DNA, form adducts with proteins (partly with inhibiting consequences ^15^), and cause oxidative damage in the cell by depleting thiol pools ^15^. 5-nitroimidazoles are in fact prodrugs, which have to be reduced at their nitro groups in order to become toxic. This reaction, however, takes place quantitatively only in microaerophilic/anaerobic organisms and has been suggested to be catalyzed by several enzymes and factors such as ferredoxin ^16^, nitroreductase ^17^, and thioredoxin reductase ^15^. Resistance to 5-nitroimidazoles in clinical Tv isolates does occur, sometimes leading to extended and complex treatment regimens ^18^. Clinical metronidazole resistance is based on decreased oxygen scavenging in the cell, leading to higher intracellular oxygen concentrations ^19^. Accordingly, expression of flavin reductase 1, an enzyme that uses flavin mononucleotide (FMN) to reduce molecular oxygen to H _2_O _2_, has been described to be downregulated or even shut-off in metronidazole-resistant Tv ^20, 21^. In addition, a correlation between metronidazole resistance and mutations in the genes for nitroreductase 4 and 6 was found ^22^.

Due to the occurrence of Tv strains refractory to 5-nitroimidazole treatment, the search for alternative treatments has never stopped. In recent years, a number of promising alternatives were presented, including pentamycin ^23^, boric acid ^24^, N-chlorotaurine ^25^, and drug-free chitosan ^26^, all of which would have be to administered intravaginally and not systemically, as is the case with 5-nitroimidazoles. Further, a combination of metronidazole and miconazole, administered intravaginally, was shown to greatly reduce adverse side effects often reported for systemic metronidazole treatment ^27^.

## Pathogenicity

The last few years have seen a number of major advances in our understanding of Tv pathogenicity. In a number of studies, including proteomic and glycobiological approaches, several key components of the Tv cell surface were described. First, a detailed chemical structure of Tv lipoglycan, a surface molecule strongly binding to human galectin-1 and -3 ^28^, was published ^29^. Further, a large surface proteome study was performed ^30^, identifying 261 putative membrane proteins, including ABC transporters and 11 BspA proteins. BspA proteins constitute a huge surface protein family in the Tv genome comprising 911 members ^31^. They could bind to proteins of the extracellular matrix of the host epithelium, *e*. *g*. fibronectin, and elicit strong immune responses. In addition, this proteomic study revealed the existence of two hypothetical proteins which seem to enhance adhesion of Tv to the host epithelium ^30^. Another proteomic study was performed using exosome-enriched cellular fractions of Tv, leading to the identification of 215 proteins, putatively localizing to exosome vesicles ^32^. Among these proteins were one BspA-like protein and one tetraspanin. Tetraspanins are a protein family known to be involved in cell adhesion, and proteins that had before been suggested to be involved in adhesion of Tv to the epithelium, such as glyceraldehyde 3-phosphate dehydrogenase ^33^, enolase ^34^, succinyl-CoA synthetase ^35^, and GP63 protease ^36^. Importantly, a large-scale transcriptomic deep sequencing study (RNAseq) with Tv during early infection performed by another work group corroborated many of these observations ^37^. Exosomes also contain large amounts of short RNA molecules (25–200 nucleotides) and enhance adhesion to vaginal ectocervical cells (VECs) when added extraneously to Tv strains with poor adhesion capacity ^32^. It is important to note that cell adhesion is an absolutely necessary prerequisite for the lysis of host cells by Tv ^38^. After cell adhesion has taken place, several factors are assumed to be involved in host cell lysis, including metalloproteases ^39, 40^, cysteine proteases ^41– 43^, a rhomboid protease (TvROM1) ^44^, and phospholipase A2 ^45^. Tv also secretes a migration inhibition factor (TvMIF) ^46^ which can replace human migration factor (HuMIF) to trigger proinflammatory cytokine release. Possibly, this contributes to the increased risk of developing prostate cancer in Tv-infected men ^3^.

The detection of tetraspanins in Tv exosomes prompted further research on this protein family ^47, 48^. Of the tetraspanins studied, all but one (TvTsp2) were strongly upregulated upon contact with VECs ^48^. TvTsp6 changes its localization in the cell upon VEC contact and migrates from the flagellum to the plasma membrane. The C-terminal tail was found to be necessary for correct localization. Intriguingly, one tetraspanin, TvTSP8, seems to mediate Tv aggregation rather than VEC adhesion ^48^. Contact with VECs also triggers a reorganization of the actin cytoskeleton and enables the rapid transition of flagellate to amoeboid morphology ^49^. This process is mediated by TvFIM1, the only fimbrin found to be expressed in Tv.

**Figure 1.**
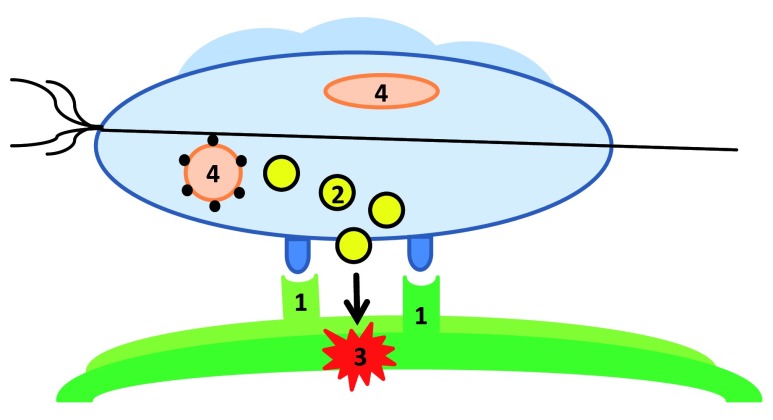
Model of *Trichomonas vaginalis* (Tv) pathogenicity. In order to exert a cytopathic effect, it is necessary ^37^ that Tv (light blue) binds ( **1**) to the extracellular matrix (light green) or the host epithelium (dark green). Binding is accomplished by several surface proteins and other surface molecules that bind to a structure on the host’s cell surface. Among these are lipoglycan ^27^, BspA ^28, 30^, tetraspanins ^28, 45, 46^ and several others, such as glyceraldehyde 3-phosphate dehydrogenase ^32^, enolase ^33^, and succinyl-CoA synthetase ^34^ on the Tv surface, galectins-1 and -3 ^28^ on the host cell surface, and fibronectin ^32^ in the extracellular matrix. Several Tv factors necessary for adhesion to the host epithelium reach the Tv surface or the epithelium surface *via* exosomes ^31^ ( **2**). Damage to the host cell is caused by several effectors ( **3**), including cysteine proteases, metalloproteases, rhomboid proteases, and phospholipase A2. Tv migration inhibition factor might favor the development of neoplasia in the prostate ^44^. In the presence of *Mycoplasma hominis* ^48^ and Tv virus ^49^ ( **4**), symptoms might be exacerbated.

When discussing the pathogenicity of Tv, it is also important to take into account other microorganisms that coincide with the parasite, especially *Mycoplasma hominis* and TVV. In the presence of *M*. *hominis*, Tv infection triggers a far more pronounced proinflammatory reaction than in its absence ^50^. The enhancing effect of TVV (which resides in about half of all Tv isolates) on the proinflammatory response seems to be even stronger ^51^, as TVV is sensed by Toll-like receptor 3 on the surface of VECs. Especially worrying is the observation that metronidazole treatment, accompanied by the release of large amounts of virus particles from necrotic Tv, further amplifies this adverse response. The contents of this section are visualized in Figure 1.

## Biochemistry and cell biology

The last few years have seen several transcriptomic and proteomic studies addressing the impact of growth and culture conditions on gene expression in Tv. Deep sequencing of RNA libraries was applied to identify genes that are differentially expressed under oxidative stress ^37^ and glucose restriction ^52^. Oxidative stress led to an upregulation of expression of 218 genes after 2 hours, including peroxiredoxins (Prx), thioredoxin reductase, thioredoxins, superoxide dismutases (SODs), rubrerythrin, and ferredoxins ^37^. Upregulation of SOD and Prx upon oxidative stress at the protein level had already been reported before ^53^, underpinning the validity of the transcriptomic approach. Interestingly, glucose starvation also led to upregulation of SOD, Prx, and rubrerythrin, resulting in a more H _2_O _2_-resistant phenotype ^52^. Most glycolytic enzymes, however, were downregulated in glucose-starved cells, accompanied by a strong upregulation of glutamate dehydrogenase, which produces α-ketoglutarate by oxidative deamination of glutamate. Also, autophagy was observed in glucose-starved cells, and autophagy markers, *i.e.* autophagy-related genes (atg), were upregulated in expression ^52^. In a phosphoproteomic study, 82 phosphoproteins were discovered in Tv ^54^, a number conspicuously low given that more than 1000 genes for kinases exist in the Tv genome ^54, 55^.

The glycobiology of Tv has received considerable attention recently, and a comprehensive study on N-glycan composition in four Tv strains was published ^56^. In all strains, a major core structure, a truncated oligomannose form (Man5GlcNAc2) with α1,2-mannose residues, could be identified. In contrast, modifications with phosphoethanolamine and terminal N-acetyllactosamine varied depending on the strain studied. Moreover, the core structure is often decorated with xylose ^29, 56^, which has been described as typical for trematodes and plants. Indeed, Tv encodes a functional UDP-xylose synthase ^57^, the first to be described in a unicellular parasite. Further, asparagine-linked N-glycans of Tv were found to bind human mannose-binding lectin and retroviral lectins ^58^.

Naturally, the hydrogenosome, as a model for organelle evolution, has remained one of the major focuses in the Tv field. Again, proteomic studies provided a deeper insight into hydrogenosome biology. A study on hydrogenosomal membrane proteins, for example, demonstrated that hydrogenosomes and mitochondria have important core membrane components in common which are responsible for protein import and metabolite transport ^59^. Hydrogenosomes also contain a dynamin-like protein which is likely to be involved in hydrogenosomal fission ^60^. Nevertheless, essential differences with mitochondria also exist which can be attributed to the microaerophilic lifestyle and evolutionary adaptations of Tv and other related parasites. This is also reflected in the much lower number of proteins in the hydrogenosome ^61^ as compared to mitochondria, *i*. *e*. about 500 vs. 1000–1500. The proteome’s composition is also rather variable, as the expression levels of many hydrogenosomal proteins were found to depend on available iron concentrations ^62, 63^. This is in line with the high abundance of iron-sulfur cluster proteins such as pyruvate:ferredoxin oxidoreductase, hydrogenase, and ferredoxin in this organelle. Unfortunately, it is hard to predict the localization of proteins to the hydrogenosome based on sequence information alone because protein import seems to depend on as yet poorly defined internal sequences, rather than on N-terminal targeting sequences. The latter seem, if at all existent, to be dispensable in most cases, likely due to the loss of the electrochemical gradient ^61, 64, 65^. This difficulty can, however, be partly overcome by applying sophisticated machine learning approaches ^66^. Also, other recent findings are difficult to put into perspective, *e*. *g*. the obvious functional redundancy of one of the most abundant proteins in the hydrogenosomal membrane, Tvhmp23 ^67^, or the localization of arginine deiminase to the hydrogenosome while other key enzymes of the arginine dihydrolase pathway reside in the cytosol ^68^.

## Genomics and gene expression

The Tv genome is extremely large for a protist and might be even larger than originally anticipated, *i*. *e*. 175 Mb in size ^69^ rather than 160 Mb ^6^. Intriguingly, as much as 65% of its content consists of repetitive sequences, including transposable elements such as representatives of the types Maverick and Tc1/mariner ^70^, and microRNA ^71^. The expansion of gene families is a common phenomenon in Tv, so that the vast number of 60,000 genes has accumulated in the genome ^6^. On the other hand, the proportion of pseudogenes seems to be extraordinarily large as well, with, for example, as much as 46% of the 123 transmembrane adenylyl cyclases being truncated or having nonsense mutations ^72^. However, many pseudogenes are being transcribed, leading to a high representation of pseudogene mRNA in the long non-coding RNA pool ^73^. In total, only about half of the annotated genes are being expressed but almost all gene families are represented ^73^. It is likely that Tv harnesses this fluctuant nature of its genome to adaptive innovation, *i*. *e*. evolution. This flexibility might apply to annotated, functional genes as well. For example, seven full-length isoforms of the enzyme flavin reductase (FR1-7) with varying relatedness to each other are present in the genome ^21^, but only FR1 has a Km for FMN which is low enough to be of plausible physiological importance. Three other FRs have high Vmax but also high Km, and the remaining three have low Vmax and high Km. Nevertheless, all of the less specific isoforms are expressed, if not in all strains, and can, at very high expression levels, partly substitute for FR1.

The last few years have also seen several advances in our understanding of gene expression in Tv. Especially well studied are the Myb-like transcription factors tvMyb1-3, which are known to bind to the promoter sites MRE-1/MRE-2r and MRE-2f of the hydrogenosomal malate dehydrogenase gene, also known as ap65-1 ^74^. In the case of tvMyb3, the DNA-binding site was crystallized and its structure determined ^75^. In a suite of excellent studies, the same group also revealed the mechanism of nuclear import of all three tvMybs ^76– 78^. Further, core promoter elements in Tv ^79^ and polyadenylation signals ^80^ were described. Finally, Tv mRNA was found to possess a metazoan/plant-like cap structure and a metazoan/plant-like capping enzyme ^81^.

## Conclusion

In recent years, considerable progress was achieved in the Tv field. Although there are still many open questions regarding Tv’s epidemiology, particularly in the context of facilitated HIV contagion and cancer, our understanding of Tv’s pathogenesis made a large leap forward and the picture is becoming ever more complete. In the treatment of Tv, several interesting alternatives, especially topical treatments, might eventually replace metronidazole, which potentially has worrying side effects. Finally, the genome of Tv has remained a fascinating colossus, whose complexity will trigger plenty of further research in the years to come.
